# Technology adoption and diffusion in healthcare at onset of COVID-19 and beyond

**DOI:** 10.1177/08404704211058842

**Published:** 2022-03-03

**Authors:** Lili Liu, Antonio Miguel-Cruz

**Affiliations:** 1 8430 University of Waterloo, Waterloo, Ontario, Canada.; 2 University of Alberta, Edmonton, Alberta, Canada.; 3 Glenrose Rehabilitation Hospital, Edmonton, Alberta, Canada.

## Abstract

This article presents an overview of the effects of the COVID-19 pandemic on the adoption and diffusion of technologies including within healthcare. Consumer technologies have been rapidly applied to mitigate negative health impacts such as social isolation, or to monitor the health and function of family members separated by quarantine. As the lines between consumer technologies and professional health technologies blur, there is an opportunity to examine the outcomes of accessible and familiar technologies used by consumers. The rapid diffusion of technology uptake challenges traditional frameworks that describe technology acceptance and adoption. There is an opportunity to understand the impact of experience of use and involuntariness on technology diffusion. Beyond the onset of the pandemic, the management of post-COVID syndrome, which some see as the next public health crisis, is an opportunity to accelerate the diffusion of home monitoring technologies already benefiting people living with other chronic health conditions.

## Introduction

The COVID-19 global pandemic has accelerated the adoption of technologies in all sectors of society, including education, business, healthcare, and social interactions. During lockdowns, businesses, schools, public transportation, places of worship, and public spaces were shut down, along with quarantine of individuals within a social “bubble.” This resulted in people resorting to the Internet to carry out their roles and responsibilities related to their employment, looking after others, and socializing. Usage of on-line conferencing platforms such as Zoom increased 10-fold.^1^ Some cities, such as Bangalore, saw Internet traffic increase by 100%.^2^

In 2019, 67% of the world was using mobile devices, and of these, 65% were smartphones. The fastest growth was in sub-Saharan Africa.^3^ In the same year, 204 billion apps were downloaded,^3^ and as of January 2020, 3.8 billion people subscribed to social media.^3^ Since the lockdowns in March 2020 following the pandemic onset, Canadians increased on-line spending especially on technology products. For example, 44% purchased technologies such as computers, laptops, and tablets. Similarly, 40% purchased smartphones and 42% acquired on-line video streaming services. A third, or 34%, of Canadians also increased spending on home and mobile Internet connections.^4^

According to the Canadian Perspectives Survey Series nearly half of Canadians (46%) increased their use of free streaming video services such as YouTube.^4-7^ The increase was most evident among persons aged 15 to 34 years old, with just over two-thirds (68%) reporting increased use of on-line video streaming services. Canadians also reported increased use of free on-line information services and on-line educational services since the pandemic began.^4^

### Consumer technologies for health

At the same time, the line between healthcare devices and consumer products, such as smartphones, smart watches, and fitness trackers, is blurring. Despite limited evidence on the validity of using consumer products for health applications (see Lapierre et al.^8^ on fall detection devices, and Neubauer et al.^9^ on devices for dementia-related wandering), consumers are pushing the boundaries and raising expectations for these ubiquitous consumer products to meet their everyday needs in relation to management of their health-related activities.^10^ Over time, more consumer products will be incorporated into health outcomes research. For example, in a study that examined the effect of the pandemic lockdown on the mental health of long-term care residents,^11^ researchers showed how “mitigating strategies,” such virtual visits using technology, prevented worsening of mental health outcomes^12^.

### Technology adoption in healthcare

At the onset of COVID-19, healthcare professionals and service providers quickly incorporated existing technologies and platforms to deliver care and home monitoring to manage risks of COVID-19 transmission associated with in-person interactions^1,13-15^. Although telehealth (or telemedicine and telerehabilitation) sites have been in place for over two decades in Canada, virtual interactions became a preferred replacement for in-person medical appointments which became less feasible. With virtual visits, patients could continue to access quality care without exposing themselves to the virus.^16^ Although originally intended to address geographic barriers for patients in remote or rural communities, telehealth now addresses the challenges created by public health measures to avoid physical contact between healthcare providers and patients.

According to Canada Health Infoway, primary care visits in Canada conducted virtually with telehealth technologies jumped from 4% to 60% at the onset of COVID-19.^10^ Support for virtual care is evident among recipients as well as providers of healthcare. Indeed, even prior to the COVID-19 pandemic, two-thirds of Canadians stated they would use virtual care if it was offered through their benefit plans.^16^ Although 59% of Canadians prefer to speak to their doctors in person, 84% of telemedicine users would continue to use it after the pandemic is over.^17^

For primary care during COVID-19 in Ontario, there was an 80% decrease in office visits and a 56-fold increase in virtual visits beginning in mid-March 2020.^18^ Virtual care made up 71% of all visits. As expected, the highest proportion consisted of individuals with high healthcare needs (73%) and the lowest proportion consisted of children (57.6%).^18^ In Ontario, mass adoption of virtual care using “any type of technology” such as telephone calls, commercial videoconferencing software (Apple FaceTime, Microsoft Teams, and Zoom) and apps (WhatsApp) has been facilitated by temporary billing codes introduced on March 14, 2020^16,19^. These codes allow physicians to be reimbursed at the same rate as in-person visits and “have been continued indefinitely”^19^. During the first six months of 2020 in Ontario, most residents received at least one virtual visit with their physicians. Older age and lower income do not seem to be barriers to receiving virtual care.^19^

Although these billing codes meet immediate needs and are an acceptable way to receive services and deliver care, the codes do not cover all services. The billing codes are only used to compensate general practitioners for brief patient visits and assessments, specifically for minor assessment, mental healthcare or counselling, and specialist interaction. More complex services are not adequately compensated.^16^

Indeed, the COVID-19 pandemic hastened the adoption of everyday technologies like Internet usage, smart phones, social media, and videoconference services. Pre-pandemic, some of these everyday technologies were used by a segment of the population but during the pandemic, they became a must-have for many so that they could carry out routine life activities—working, grocery shopping, going to school or post-secondary education, socializing, attending recreation and religious activities, and, of course, obtaining healthcare.

## Technology adoption framework: Adoption and diffusion

This rapid adoption of technologies in everyday life on a global scale is unprecedented. Evidence of the rapid adoption of technologies can be seen in the diminishing time span for technology diffusion (measured as the percentage of users with access or adoption of a technology over time). For example, mobile phones, smartphones, and wearables devices show fast-rising adoption rates that went from nearly 0% to 50% adoption in about five years.^20^ This is a remarkably fast rate of adoption if we consider that the landline telephone took 50 years to achieve 50% adoption rate^21^.

**Figure 1.**
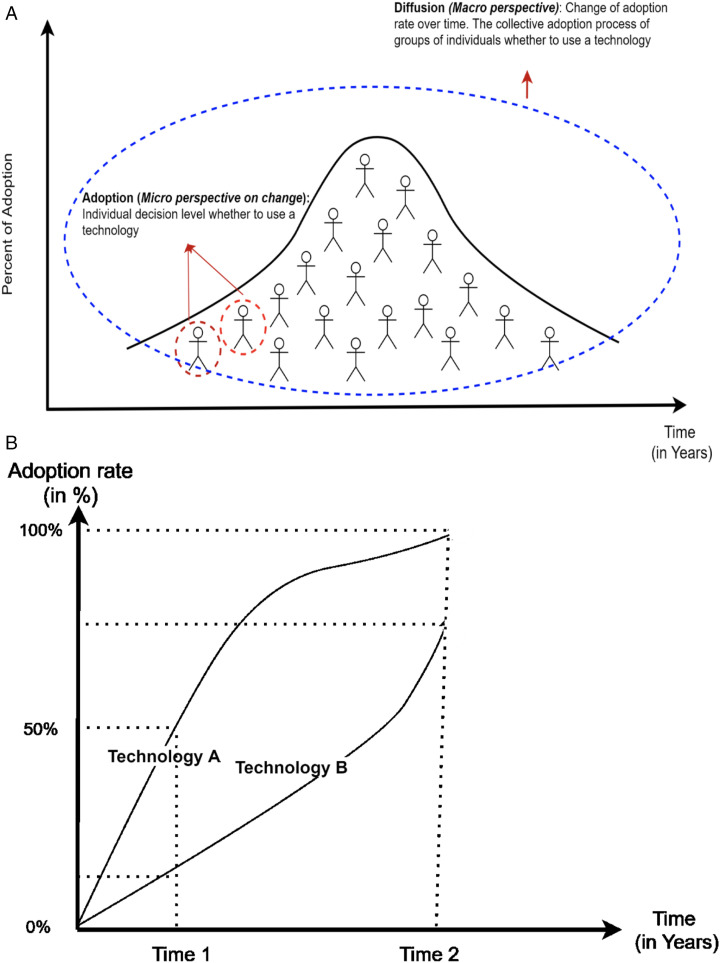
(A) How individual adoptions shape the diffusion curve. (B) Relationship between adoption rate and diffusion of technology. Diffusion describes the adoption process in a population over time. The adoption rate of technology A is higher than B.

Simply put, *adoption* and *diffusion* refer to the processes for the spread of a new idea, technology or innovation over time^22^. Technology adoption refers to one’s decision to use a technology on an individual level. Technology diffusion describes the collective adoption process of groups of individuals who use a technology over time. Thus, the adoption process can be seen as a *micro* perspective on change (ie, individual behavioural change to use a technology), whereas diffusion is a *macro* perspective, and describes how technology use spreads through a population (see Figure 1).^22,23^ The rate of individual adoption over time shapes the population technology diffusion curve. Figure 1 shows a hypothetical relationship between adoption rate and diffusion for two technologies, named “A” and “B”. On the *Y*-axis is the adoption rate (measured as the percentage of new users to all potential users that has adopted a technology), on the *X*-axis, is the time of adoption (in years or months). It is apparent that Technology A exhibits higher adoption rate than Technology B over the same period of time. At Time 1, Technology A was adopted by 50% of its target population, whereas Technology B was adopted by just over 10% of its target population. By Time 2, all potential users have adopted Technology A, whereas the adoption rate for Technology B was 75%.

There is a set of theories used to explain acceptance and adoption of technologies. The *Technology Acceptance Model,*^24^ the *Innovation Diffusion Theory,*^25^ and the *Unified Theory of Acceptance and Use of Technology*, in its versions UTAUT,^26^ and UTAU2^27^, are the most known and used. These theories share basic assumptions and three characteristics that influence technology adoption. The first assumption is that the adoption process is not a single event. Instead, it is a complex process in which an individual’s beliefs and attitudes are formed over time and lead to a final decision about whether to adopt a technology^23^. Second, most technology adoption can be accurately predicted from an “appropriate measure” of the *individual’s intention* to perform the behaviour in question, in this case, technology use or adoption^22^. In technology acceptance and adoption theories, these appropriate measures are the systems of individuals' beliefs. The most common systems of beliefs are the degree of ease associated with a technology, whether the technology will help the end-user to attain gains in performance (usefulness), whether significant others believe the individual should use the new technology (social influence), and whether organizational and technical infrastructure exist (facilitating conditions) to support use of the technology.^26^ When facilitating conditions are not created, users can perceive there are barriers toward adoption. Canadian health insurance billing regulations within individual provinces and territories contribute to the largest barrier to the widespread adoption of virtual care in routine office-based practice^28,29^. In terms of characteristics, the theories take into account the individual characteristics, innovation and contextual aspects that determine the adoption of technologies.^22^ Individual characteristics include sex, age, and experience of use. Innovation takes into account whether the technology “is perceived as being better than its precursor”^30^ (also called relative advantage). Finally, contextual characteristics take into account whether there is a policy in place that foster voluntariness toward the use of the technology.

Health leaders can take several measures to enhance technology adoption. First, health leaders should make available evidence on the effectiveness of a specific technology through clear communication with end users such as practitioners and patients. If evidence is not yet available, as is the case for many emerging technologies, health leaders can incorporate a process for monitoring and tracking key performance indicators in order to contribute to the body of evidence. For example, our experience from previous studies suggests that, in healthcare settings where use of new technologies was not mandatory, rehabilitation therapists’ decision to adopt a technology was most influenced by whether they believed a technology can help them achieve treatment goals with their clients.^31^ In the same study, we found that facilitating conditions was the strongest salient for use of new technologies among rehabilitation professionals.^31^ Thus, healthcare managers who identify and address facilitating conditions would help ensure that new technologies are adopted. Facilitating conditions include technology support personnel, and easy-to-use platforms for scheduling and booking time for use of a technology. If bespoke technologies are designed for specific interventions, such as software for serious games, end products are more likely to be successfully adopted if users, that is, health practitioners and clients, are included early in the co-design process.

### Role of experience and voluntariness

The role of *experience of use* and *voluntariness* toward the adoption of a technology are variables included in theories such as UTAUT and UTAUT 2 and in TAM2. The experience of use of a technology has been operationalized as *high* or *low* experience, or in the number of years of use of a technology.^26^ The voluntariness toward the adoption of a technology is understood as “the degree to which use of the innovation is perceived as being voluntary or free will”^30^. Traditionally, experience of use and voluntariness have been introduced in the theories as *moderator variables*. A moderator variable can make an association between variables stronger, weaker, or even disappear. For example, the UTAUT posits that the influence of ease of use on technology acceptance is moderated by user experience, such that the effect will be stronger for users with less experience. This means that if a novice user finds a technology difficult to use, the user may abandon the technology earlier compared to a more experienced user. UTAUT also posits the predictor effect of social influence on technology acceptance will be moderated by voluntariness and experience of use of the technology, such that the effect will be stronger particularly in mandatory settings among users at early stages of experience of technology use (an inexperienced user, or a user who is mandated to use a technology, is more easily influenced by others such as colleagues). As experience of use and voluntariness toward the adoption of a technology have been explored to a much lesser extent,^32^ we have a once-in-a-lifetime opportunity to conduct empirical studies aimed at examining the moderator effects of these variables on technology adoption on a global scale with a wide range of consumer products and health technologies. At no other time in history could we examine adoption and diffusion of technology in healthcare (and in social interactions and entertainment) in a way that we can now as technology use was, in general, involuntary at the onset of and during the pandemic. The rapid diffusion of some technologies on a global scale also means that larger numbers of populations are now more experienced with use of these technologies.

Since the onset of the pandemic, the voluntariness of technology adoption in healthcare has been influenced more by necessity to provide basic services to patients, and less by the traditional technology procurement process used by health leaders. In other words, the “requirement” to use technologies such as information communication technology, to conduct virtual visits between patient and health professional could be considered involuntary, and mandated by the public health restrictions of the pandemic, not by healthcare administration. This is an opportunity for health leaders to examine impact of the moderator effects of this “involuntary” use and development of “experience” during the rapid technology diffusion imposed by the pandemic. Health leaders could advocate for continuation of practice approaches that show gains in efficiency as a result of technology diffusion during the pandemic.

## Technology adoption in healthcare beyond COVID-19

The involuntariness of use of technologies at the onset of COVID-19 that created a rapid pace of technology diffusion among populations places less importance on the level of individual “acceptance” of technologies which typically would precede adoption. During mandated physical distancing, instead of whether an individual accepted videoconferencing, the question became which video conference platform users preferred, and which were acceptable by healthcare systems.

Some health practitioners and patients are concerned that the pace of technology diffusion will decrease or reverse if facilitating conditions, such as reimbursement codes for physicians to provide virtual care, are removed. Several aspects of healthcare benefit from the rapid diffusion of technology at the onset of COVID-19. An important aspect is in home health monitoring. During the onset of COVID-19, Italian researchers used a cloud-based patient management platform featuring “clinical-grade continuous and spot-checking measurements, digital care pathways, and remote patient surveillance”.^33,34^ Remote monitoring of patient measures, such as oxygen saturation, allowed for 80% of their 200 patients to be remotely cared for.^33,34^ Between November 2020 and January 2021, 70% of admissions related to COVID were directly from home to hospital without going through the emergency department.^33,34^ An important feature of this platform was that patients wore a bracelet that provided continuous data to emergency medical services, the general practitioner’s team and the hospital’s chronic care team. Despite living in a rural community, patients did not feel alone.

Remote home monitoring is necessary for the increasing numbers of patients experiencing Post-COVID Syndrome (PCS) (also known as chronic COVID syndrome, long COVID syndrome, post-acute COVID-19 syndrome, and long haul COVID-19^35-38^). It is believed that PCS is the next public health crisis, similar to other chronic conditions like diabetes and COPD.^39,40^ The multisystem disease is associated with experience of symptoms for one to six months, or a slow pace of recovery, after the acute phase of COVID-19.^37-39,41^ The most common symptoms are shortness of breath, fatigue, ageusia and anosmia.^40^ Ten to 35% of individuals experience this syndrome; it can reach up to 85% in hospitalized individuals.^41^ Patients recover slowly with rest and symptomatic treatment with gradual increase in activity. Prolonged symptoms are associated with difficulties sleeping, anxiety, or depression.^42^ In a prospective study of the first 100 consecutive patients, PCS was found to include “brain fog”, headache, numbness and myalgias.^36^ Despite mild symptoms in the acute stage of COVID-19, some patients experienced persistent and debilitating brain fog and fatigue, affecting cognition and quality of life over the long term due to inability to carry out physical labour and simple activities of daily living, causing them to be dependent on family. Many become depressed or anxious when they become unemployed.^43^

Home monitoring is not new in healthcare, but post-COVID syndrome provides an impetus for healthcare to diffuse technologies that address the large wave of populations that will require support to overcome the chronic and debilitating symptoms. Ubiquitous commercial technologies (such as telephone and video conference platforms) covered by billing codes are necessary for patients to receive care from their homes while preserving their energy to recover. Mobile devices necessary for patient monitoring need to be accessible and affordable. Hence, health leaders could advocate for policies that make telecommunication costs more affordable in Canada.

Health leaders who support home health monitoring have also raised concerns that monitoring technologies are not considered medical devices and thus are not subject to government regulation, or have not yet received approval for medical purposes.^44,45^ In addition, approval of medical devices for use at home does not necessarily indicate that the devices are safe or effective for monitoring, nor that the risks and benefits are studied.^44^ In terms of privacy and security, the main concerns lie on whether data are anonymized, whether the users have sovereignty over data (ie, opt out of participating and can stop sharing their home monitoring data at any time), and whether commercial use of data is banned^44^. Best practices for privacy and security should inform policies that ensure industries: (a) test their products as rigorously as possible, by conducting clinical- and non–clinical performance and human-factors testing to demonstrate that their products are safe and effective,^44^ (b) adopt a systems view rather than just a product view,^44^ and (c) develop ethical guidelines tailored explicitly to home monitoring technologies (ie, to practice “ethics by design”).^44^ From a systems view, developers take into account the context in which the home monitoring technology will be deployed, analyze the additional challenges that need to be overcome and take the appropriated measures to guarantee a successful implementation.^44^ Ethics by design means that an organization that develops home monitoring has in place policies that establish ethical norms of behaviour, prompt people to think about ethics routinely, and help people recognize ethical conduct and adjust behaviour accordingly.^46^

While there are opportunities associated with the adoption and diffusion of technologies that enable virtual healthcare, legal experts caution policy-makers, health service providers, and patients. According to Hardcastle and Ogbogu,^47^ virtual care should prioritize continuity of care using consistent care providers who have custodianship of one’s health data, and should be used for appropriate types of healthcare assessments and interventions. The type of platform should also ensure that patient data is secure and confidential.^47^ For example, during the early phase of the pandemic when billing for virtual care included use of “any type of technology,” Zoom was hacked which revealed risks to patients' privacy.

## Conclusion

The onset of the COVID-19 pandemic in early 2020 was associated with a global diffusion of everyday technologies. Early evidence indicates that, although involuntary for many, the majority of users have been satisfied with the use of technologies in their everyday lives, including as a way to receive healthcare services from general practitioners and mental health service providers. The benefits of efficiency, reduced travel, and better access to health services warrant serious consideration by health leaders to maintain virtual access as part of the new status quo. The rapid diffusion of technologies also provides an opportunity to examine the factors that moderate users' adoption of technologies, particularly as they relate to users' prior experience and perceived voluntariness to use technology. Unlike the past, the pandemic created a new condition where the impact of personal choice to adopt technology was less important than the impact of population diffusion of technology. It is a critical time for health leaders to identify, advocate for and facilitate technology that has shown gains in efficiency, as a new norm. Health leaders also have an opportunity to propel the diffusion of home monitoring technologies which will benefit populations with chronic health conditions, including persons living with post-COVID syndrome. However, successful technology adoption and diffusion must be accompanied by principles that ensure data security and patient privacy.
